# Function and evolution of sex determination mechanisms, genes and pathways in insects

**DOI:** 10.1002/bies.201000043

**Published:** 2011-01

**Authors:** Tanja Gempe, Martin Beye

**Affiliations:** Department of Genetics, Heinrich Heine UniversityDuesseldorf, Universitaetsstrasse 1, 40225 Duesseldorf, Germany

**Keywords:** evolution, evolutionary forces, gene duplication, sex determination, sexual differentiation and selection

## Abstract

Animals have evolved a bewildering diversity of mechanisms to determine the two sexes. Studies of sex determination genes – their history and function – in non-model insects and *Drosophila* have allowed us to begin to understand the generation of sex determination diversity. One common theme from these studies is that evolved mechanisms produce activities in either males or females to control a shared gene switch that regulates sexual development. Only a few small-scale changes in existing and duplicated genes are sufficient to generate large differences in sex determination systems. This review summarises recent findings in insects, surveys evidence of how and why sex determination mechanisms can change rapidly and suggests fruitful areas of future research.

## Introduction

The regulation and evolution of sexual development have long been of central interest in developmental and evolutionary biology. One key question has been how sexual fate is determined and regulated, giving rise to the sexually dimorphic traits that play such a dominant role in animal evolution and behaviour. Much has been learned about how sex determination is realised and is integrated into the developmental program from model systems; the insect *Drosophila melanogaster*, the nematode *Caenorhabditis elegans* and the mouse *Mus musculus* 1–4. These studies have not resolved the question of why the regulatory principles of sex determination are so bewilderingly different and how this diversity is generated molecularly.

Sex determination mechanisms can vary substantially between phylogenetically closely-related species 5–7 and even within a single species 8–10. The same mechanism can apparently be regulated by different genes 11, 12. This implies that these mechanisms evolve very rapidly, despite the antiquity of the two sexes.

Here, we review recent advances made in insects that have begun to uncover how diverse sex determination systems are generated and regulated 12–22. The evolutionary and molecular routes taken broaden our understanding of how and why novel regulatory controls of a developmental process evolve. The key questions we address in this paper are: (i) How are differences in sex determination molecularly realized; (ii) how did differences in sex determination evolve from a pre-existing genetic repertoire; (iii) what forces drive sex determination systems to diverge?

Since the first reports of a sex determination mechanism 23 and of the inherited basis of sex determination 24, 25 – both made in insects – an astonishing diversity of mechanisms has been uncovered in a variety of species and phylogenetic lineages 5, 7, 26, 27. Considering insects, classical genetic and cytological studies have identified a variety of genetic and environmental signals that determine the two sexes. In the fruit fly *D. melanogaster*, for instance, the double dose of X chromosomes 28 determines femaleness, a single dose maleness. Other dipteran insects (*e.g. Musca domestica* and *Ceratitis capitata*) employ a male-determining Y chromosome: females are XX and males are XY 13, 29. Butterflies present the opposite scenario, possessing a female-determining W chromosome: females are ZW and males are ZZ 7. In XY chromosomal systems the number of Y chromosomes can vary substantially 30; for instance in some species of the fruit fly *Anastrepha*, females are XX/XX and males XX/Y 26. Other species employ male and female determiners with no visible chromosomal differences (*e.g*. phorid fly *Megaselia scalaris* 10, *Chironomus* 9, 31). Male scale insects and white flies (both homopterans), thrips (thysanopterans) and hymenopteran insects (wasps, ants and bees) are haploid and females are diploid 7. The genetic basis of haplo/diploidy in several hymenopteran species is complementary sex determination; males are homo- or hemizygous and females heterozygous at a single locus 32, 33. In another hymenopteran species, the parasitic wasp *Nasonia vitripennis*, sex determination is consistent with a maternal imprinting mechanism 22. Differential elimination of sex chromosomes during the first stages of embryonic division is used as a sex determination mechanism in the fungus gnat *Sciara* 26. Females develop when one paternal X chromosome is lost from the 3X:2A zygote and males arise when two paternal X chromosomes are lost. Maternally-derived signals (*e.g*. the blowfly *Chrysomya rufifacies*) or environmentally-derived signals, such as the temperature of egg incubation (*e.g*. the gall midge *Heteropeza* and the fungus gnat *Sciara*), are also utilised as sex determination signals.

**Figure 1:**
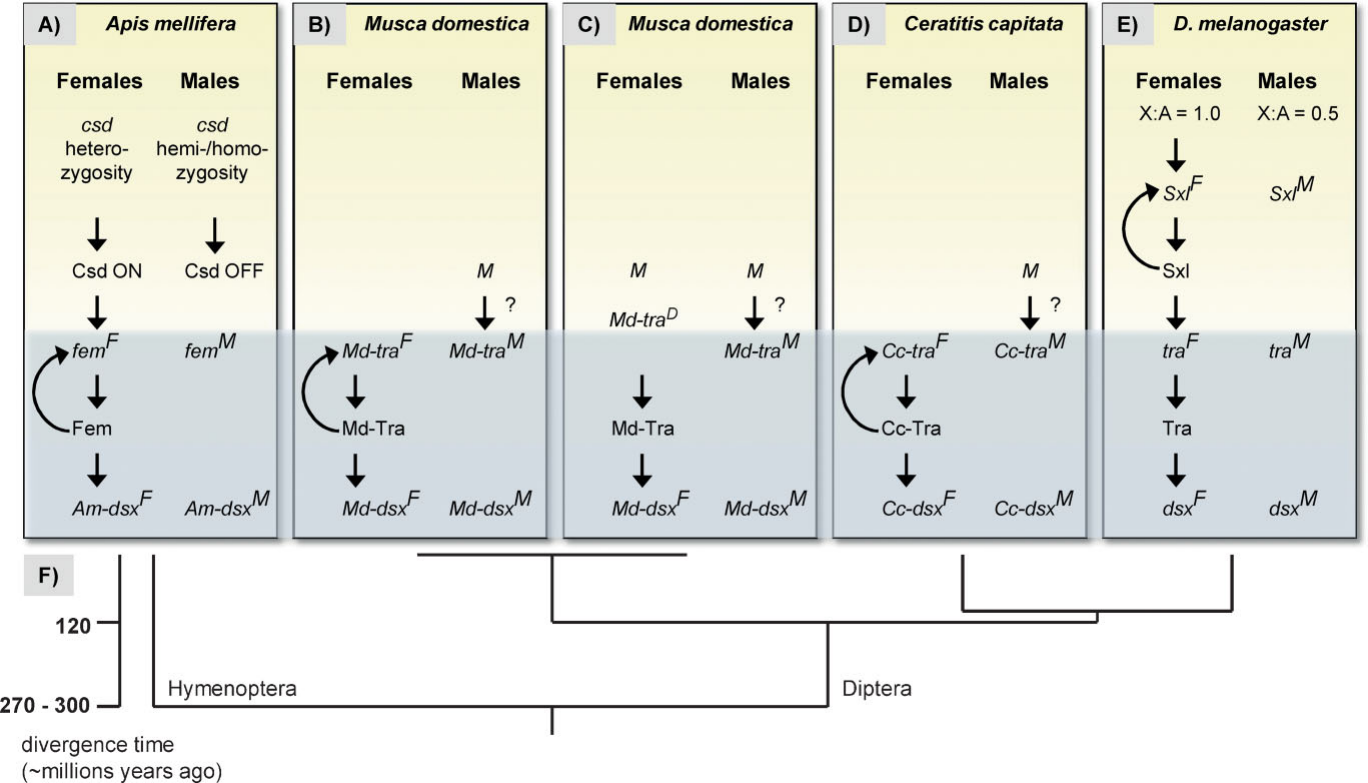
Sex determination in insect model species, with their phylogenetic relationships representing ∼300 million years of evolution. The fruit fly *D. melanogaster*, the housefly *M. domestica*, the medfly *Ceratis capitata* and the honeybee *Apis mellifera* share a common pathway (indicated by a grey box) composed of the *transformer* (*tra*) gene and its downstream target *doublesex* (*dsx*). Female spliced *tra* transcripts (*tra^F^*) give rise to Tra proteins that direct splicing of *dsx^F^* mRNAs, production of Dsx^F^ proteins and female development. When *tra* is spliced into the male variant, no Tra proteins are produced. This results in splicing of male *dsx^M^* mRNAs, Dsx^M^ proteins and male development. **A:** Sex in the honeybee (*A. mellifera*) is determined by the heterozygosity or homo-/hemizygosity of the *csd* gene. In females, different Csd proteins, derived from a heterozygous *csd* gene, direct the processing of female *fem* mRNAs (*fem^F^*) 14. The *fem* gene is apparently an orthologue of the *tra* gene 12). Fem protein regulates female splicing of *dsx*, but also self-sustains the female splicing of *fem*. In males, inactive Csd proteins that are derived from the same alleles (homo- or hemizygous *csd* genes) result in a default splicing of *fem* (*fem^M^*). (**B** and **C**) Models of two alternative sex determination systems that co-exist in *M. domestica* populations 15, 29. **B:** Sex is determined by the absence/presence of an unidentified male-determiner *M*. In the absence of *M*, maternally-derived *Md-tra* gene products establish an auto-regulative loop in females in which Md-Tra protein mediates the production of more female *Md-tra* mRNA. Presence of *M* impairs this *tra* auto-regulatory loop and also mediates the splicing of male *Md-tra* mRNAs. **C:** Sex in *M. domestica* can also be determined by a female determiner. Presence/absence of a *tra* allele, *Md-tra^D^*(=*F^D^*), determines sexual fate. In females, the presence of *Md-tra^D^* leads to female splice products and Md-Tra protein, even in the presence of the male-determiner *M* 15. In males, the male-determiner *M* mediates male *Md-tra^M^* mRNAs in the absence of the *Md-tra^D^* allele. **D:** Sex in *C. capitata* 13, 17 is determined by presence/absence of a, thus far, unidentified male-determiner *M*. In the absence of *M*, maternally-derived *Cc-tra* gene products appear to establish a *Cc*-*tra* auto-regulative loop 13. Presence of *M* mediates the splicing of male *Cc-tra* transcripts (*tra^M^*). **E:** Sex in *D. melanogaster* is determined by the dose of X chromosomes 1, 28. Double doses of X in females activate the *Sxl* gene and expression of Sxl proteins. Sxl proteins direct splicing of female *tra^F^* mRNAs that give rise to functional proteins. Sxl proteins also establish an auto-regulatory feedback loop by directing splicing of productive female *Sxl^F^* mRNAs, which maintain the female state throughout development. In addition, there is an additional feedback activity in which Tra proteins stimulate *Sxl* positive auto-regulation 98. In males, the single dose of X chromosomes does not direct early Sxl protein expression. As a consequence the downstream regulatory decisions do not occur and male *dsx^M^* is produced. **F:** The evolutionary relationship of the species used in the comparison with their approximate time scale of divergence 40, 99.

Here, we wish to limit our review to sex determination signals of the pathways that have been identified in insects. From the phylogenetic relationships of hymenopteran insects, at the base of the holometabolous insect branch (Fig. 1F), we can retrace the ancestral components that were shared by common ancestors and then explore which components evolved and established new sex determination systems.

In the next section, we summarise the different ways in which sex determination is achieved molecularly to regulate the proportions of males and females. We then turn to the question of how novel sex determination systems can evolve from the ancestral genetic repertoire, before discussing the forces that drive divergence in sex determination. Finally, we highlight potentially fruitful directions for future research.

## Regulatory diversity and common principles of sex determination

Sex determination systems use different genes and regulatory mechanisms to establish activities in either males or females (Fig. 1). These activities regulate *tra* genes, which are key, upstream components of an ancestral sex-determining pathway 12–15, 17, 20–22.

In *D. melanogaster*, the double dose of X chromosomes 28 establishes feminising activity (Fig. 1E). The X chromosome encodes several transcription factors (*e.g*. *runt*, *sisA*, *sisB*) that, through the double dose in females, activate the *Sxl* gene. Sxl proteins in females are splicing factors that splice *tra* mRNAs in females to produce Tra protein. A single X chromosome results in the absence of the Sxl protein and, as a consequence, male *tra* mRNAs with a premature stop codon are produced.

In the honeybee *Apis mellifera*, heterozygosity of the *complementary sex determiner* gene (*csd*) establishes feminising activity 33, 34 (Fig. 1A). *Csd* allelic proteins derived from heterozygous *csd* activate the *feminizer* gene (*fem*) by directing splicing to form the female *fem* mRNAs that encode the Fem protein. Proteins derived from hemizygous (haploid, unfertilised eggs) or homozygous *csd* genes are non-active. As a result, the *fem* mRNAs are spliced into the male configuration that contains a premature translation stop codon. *fem* is apparently an orthologue of the *tra* genes 12.

The housefly, *M. domestica*, exhibits a number of different sex determination systems 8, 29, 35, 36 that co-exist in this species. There is a classical system (Fig. 1B) in which a dominant male-determiner on the Y chromosome provides a masculinising activity (males are X/M-Y and females X/X; the molecular nature of *M* has yet to be identified). Other systems can have the dominant male-determiners *M* on any of the five autosomal chromosomes, and even on the X chromosome. Furthermore, a dominant female-determiner (*F*^*D*^) exists in this species that establishes feminising activity. Females are *F*^*D*^*M*/*FM* and males are *FM*/*FM* (Fig. 1C). *F*^*D*^, the dominant female-determiner, is an allelic variant of the *Md-tra* gene 15 that produces female *tra* mRNAs and active Tra protein, even in the presence of the male-determiner *M*. In the absence of the *F*^*D*^ allele, the male-determiner *M* ensures male-specific splicing of other *Md-tra* alleles and, consequently, the absence of active Md-Tra protein in males.

The medfly, *C. capitata*, also possesses a dominant male-determiner *M* on the Y chromosome that is responsible for masculinising activity 13, 17, 37 (Fig. 1D). The molecular nature of *M* is yet to be identified. In the presence of *M*, male *Cc-tra* mRNAs, but not Tra proteins, are produced. In the absence of *M*, maternally-derived Tra proteins direct splicing into productive female *Cc-tra* mRNAs.

**Figure 2:**
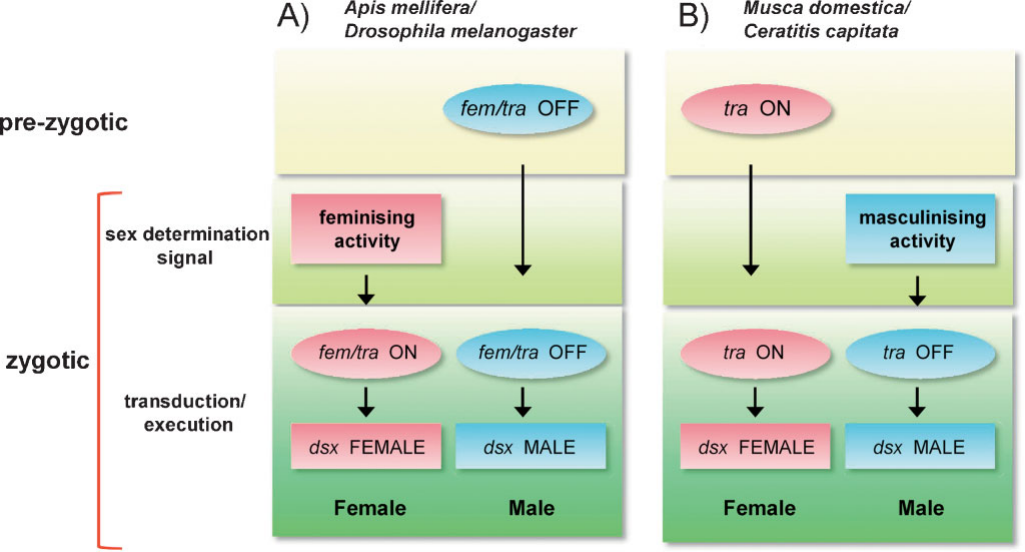
The regulatory principle underlying insect sex determination mechanisms. Species-specific, sex determination systems produce either a feminising or a masculinsing activity in the zygote to determine the two sexes in the proper proportions. In the absence of this activity, the pre-determined *tra* activity in the pre-zygote produces the alternative sex. **A:** Feminising activity in *D. melanogaster* and *A. mellifera* switches the *tra* gene from the pre-zygotic OFF (non-active) into the ON (active) state. **B:** Masculinising activity in *C. capitata* and *M. domestica* switches the *tra* gene from the pre-zygotic ON to the OFF state.

These results imply that sex determination mechanisms in insects are used in two ways (Fig. 2); sex determination mechanisms in the zygote produce either feminising activities (*D. melanogaster*/*A. mellifera*) that switch *tra* genes ON, or they generate masculinising activities (*C. capitata* and *M. domestica*) that switch *tra* genes OFF. In the absence of these signals the pre-zygotic state of *tra* (‘default’ OFF or ON) is executed, resulting in male (*D. melanogaster*/*A. mellifera*), or female (*C. capitata* and *M. domestica*) development. *tra* gene regulation in *N. vitripennis* apparently follows this latter rule 22.

How do *tra* genes implement female and male development? Tra proteins are members of SR-type splice regulators that control female splicing of the *dsx* gene (Fig. 1). In the absence of Tra proteins, male *dsx* mRNAs are produced. This regulatory principle is shared among dipteran and hymenopteran insects 12–22, 38, 39 implying that this is an ancestral principle of sexual regulation in holometabolous insects (hymenopteran insects are at the base of holometabolous insects 40). *tra* gene regulation involves a positive feedback loop in females that generates even more Tra proteins and, thereby, a stable female state throughout development 13–15 (Fig. 1). The feedback activity of *tra*, and its' role in germ cell differentiation, have been co-opted in *D. melanogaster* by the *Sxl* gene, the next upstream component 1, 41, 42 (Fig. 1), suggesting that the *Drosophila* model system is derived in these respects.

Sex-specific splicing of the *dsx* gene has been identified in other phylogenetic lineages, including the lepidopteran insects 16, 17, 43–51. The sex-specific, spliced *dsx* transcripts encode transcription factors of the DM type that have an atypical zinc-finger domain. The proteins differ in females and males at their C-terminal ends 1, which control transcription of target genes differently 52. The role of *dsx* in sexual differentiation has been demonstrated in *D. melanogaster* 52–54, *M. domestica* 16 and the lepidopteran *Bombyx mori* 55, indicative of an ancestral role in integrating sexual differentiation within the general developmental program 56–63.

*tra* genes in insects may also regulate the *fruitless* (*fru*) genes that encode a BTP zinc-finger transcription factor sex-specifically 64. *fru* specifies sexual orientation and courtship behaviour in *Drosophila* by regulating differentiation processes in the nervous system 64, 65, together with Dsx protein 66. Consistent with an ancestral role in insect sex development, *fru* sex-specific splice products have been detected in the mosquito *Anopheles gambiae* 67, *C. capitata* 17 and the hymenopteran wasp *N. vitripennis* 68.

## Evolutionary origin of novel sex determination mechanisms

**Figure 3:**
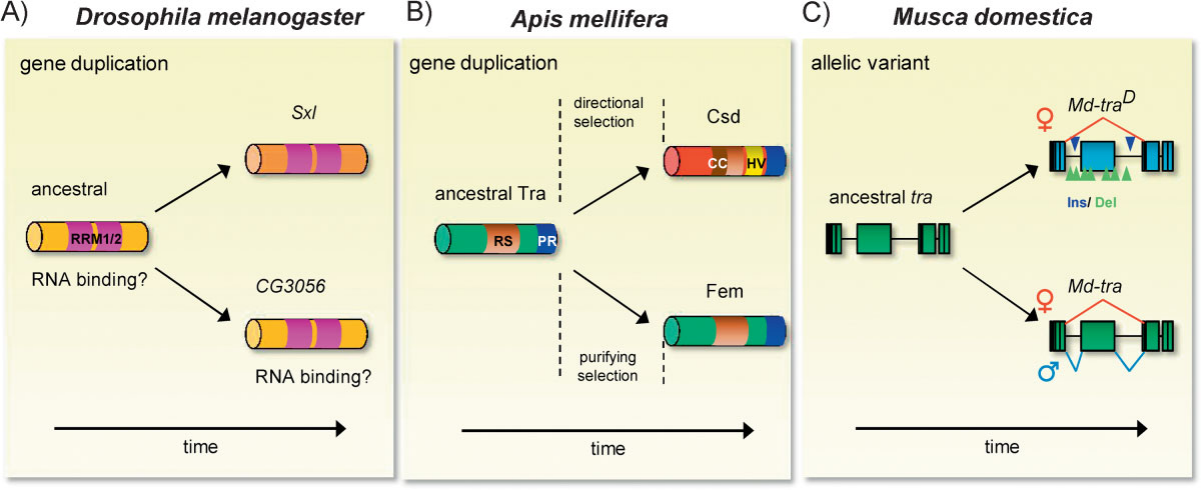
Mutational routes for the evolutionary origin of novel sex determination functions. **A:** The origin of the *Sxl* gene by gene duplication of *CG3056* in dipteran insects 69. The encoding proteins are presented schematically. RRM 1 and RRM 2 denote the two RNA-binding domains. **B:** The origin of the *csd* gene by tandem gene duplication of the ancestral *tra* gene in the *Apis* lineage. The encoding proteins are presented schematically. The evolutionary rise of the *csd* gene was accompanied by the evolution of an asparagine/tyrosine-enriched repeat that varies in number in the different allelic specificities (denoted as HV: hyper-variable region) and with the origin of a putative coiled-coil domain (denoted as CC) possibly involved in protein binding 70, 71. Adaptive evolution (directional selection) was involved in shaping the evolutionary rise of the *csd* gene 12. The paralogous sister gene *fem* evolved under purifying selection, consistent with its ancestral function 100. RS denotes the arginine/serine-enriched (RS) domain and PR denotes the proline-rich domain. **C:** The evolutionary origin of the *Md-tra^D^* allele from the ancestral *Md-tra* gene in *M. domestica* populations 15. The *Md-tra* genome structure is presented schematically. Blue triangles denote nucleotide insertions and the green triangles represent nucleotide deletions in the *Md-tra^D^* allele that lost the ability to produce the male splice variant *tra^M^* in the presence of *M*.

Functional and evolutionary analyses of sex determination genes (Fig. 1A,C,E) has revealed that small-scale mutational changes in the nucleotide sequence from existing and from duplicated copies of genes can generate novel sex determination mechanisms (Fig. 3).

The evolutionary origin of the X dosage mechanism in *D. melanogaster* is associated with the evolutionary rise of the *Sxl* gene. The *Sxl* gene encodes an RNA-binding protein that originated in the dipteran lineage by gene duplication from a copy of the ancestral CG3056 gene 69 (Fig. 3A). The regulatory relationship of *Sxl* to the sex determination cascade arose through the evolution of Sxl protein-binding sites (poly-Uridine tracts between five and eight nucleotides in length) in the *tra* gene. Signalling of the double dose of X chromosomes by transcription factors (*e.g*. transcription factors *runt*, *sisA*, *sisB*) has apparently evolved through changes in the *cis* regulatory control of *Sxl* transcription.

The complementary sex determination gene in honeybees (*Apis*) evolved through gene duplication of the ancestral copy of the *fem/tra* gene within the last 60 million years 12 (Fig. 3B). The evolutionary rise of *csd* was accompanied by the insertion of a novel hyper-variable region that consists of asparagine/tyrosine-enriched repeats 70, 71 and by single nucleotide replacement changes that produced a novel coiled-coil motif 12. The hyper-variable region is thought to play a role in the recognition process of allelic differences (*csd* alleles are only active in the heterozygous state), whereas the putative coiled-coil domain appears to encode allelic protein-binding properties (Otte and Beye, unpublished results).

The novel, dominant female-determiner *F*^*D*^(= *Mdtra*^*D*^) evolved in the housefly, *M. domestica*, from multiple small deletions and insertions in intron sequences of the *tra* gene 15 (Fig. 3C). The *Md-tra*^*D*^ allele is constitutively spliced in the productive female mode in the presence of a male-determiner *M* (Fig. 1C). *Md-tra*^*D*^ is a natural variant of the *tra* gene in housefly populations 29, suggesting that this mechanism originated recently.

These molecular analyses reveal how nucleotide changes in duplicated copies of genes and allelic variants can produce new sex determination genes and diverse mechanisms. Sex-determining variants, however, initially originated from rare mutational events in single individuals in a population. To understand how and why these initially rare variants became fixed in populations and species, we have to apply population genetic principles that account for the forces of evolutionary change. Multiple fixation processes through evolutionary time have generated the diversity of sex determination systems that we see today in the insects.

## Forces driving the divergence of sex determination systems

Random genetic drift and directional (positive) selection are the evolutionary forces that can initially drive rare sex-determining variants throughout populations of any given species. Directional selection enhances the probability of fixation, given the random fluctuations of genetic variants in a population caused by genetic drift. We do not review here the body of theoretical work that has been done in the field of the evolution of sex determination, but rather focus on the empirical molecular evidence that has so far been documented.

Positive selection, driven by fitness gains in individual population members, is a plausible source for the fixation of new sex determination systems. For instance, phylogenetic surveys in hymenopteran species suggest that the complementary sex determination mechanism has been replaced in some highly inbreeding hymenopteran species (*e.g*. parasitic wasps) 22, 32, 72. Complementary sex determination under inbreeding results in large numbers of diploid males that cannot reproduce (only haploid males are fertile); suggestive of an evolutionary advantage for alternative sex determination mechanisms in this case.

Remarkably, once new sex determination mechanisms have evolved, nature does not stop generating new sex determination genes. An ongoing divergence process can be observed in several species in which alternative sex determination systems co-exist 9, 10, 15 and in lineages in which a sex determiner gene has been replaced, but not the underlying mechanism 11, 12. Recurrent, directional selection regimes that can continuously drive sex determination systems to diverge have been proposed through several hypotheses: adjustment of sex ratios, intra-locus sexual conflict, the degeneration of sex determiners, sex ratio drive 6, 73–78 and sexual selection 79–81.

The *adjustment of the sex ratio* hypothesis states that the rise of new sex determiners can be selectively advantageous in sub-divided or inbreeding populations or when male and female fitness are affected differently by environmental factors 73, 74, because this will allow adjustment for the optimal sex ratio. The *intralocus sexual conflict* hypothesis argues that a newly evolved dominant male- or female-determiner will increase in frequency when it is closely linked to a gene with beneficial effects in the sex this gene determines 75, 76. The *degeneration* hypothesis states that the cessation or reduction of meiotic recombination at primary, sex-determining loci results in a gradual loss of the efficiency of selection (for instance, the efficient removal of deleterious mutations in a population), resulting in an evolutionary degeneration of a sex determiner gene 6, 12, 82, 83. A malfunction in sex determination favours directional selection of an alternative, novel sex determiner. The degeneration hypothesis implies an evolutionary paradox: although meiotic recombination and the mixing of genetic material require two sexes, the genes that establish them may lack the evolutionary advantage of sexual reproduction. The *sex ratio drive* hypothesis suggests that X-linked, meiotic drive factors increase in frequency by inducing the loss of Y chromosome-containing sperm 77, 78. There is, thus far, no direct evidence that any one of these directional selection scenarios favoured the rise of novel, sex determination systems. These hypotheses are also difficult to test, as it is not known whether fitness gains or losses currently observable arise from novel sex determiner genes themselves, or are associated with other, secondary effects (for instance, suppression of recombination around the sex determination gene or accumulation of sex-specific beneficial alleles) 84.

The function of sex determination hierarchies (Fig. 1) is inconsistent with the view that sex determination systems evolve because of sexual selection. The sex determination signals of *M. domestica*, *C. capitata* and *A. mellifera* all regulate *tra* genes that control sexual development in its entirety (Fig. 1). They do not specify a particular set of sexually-dimorphic traits on which sexual selection can operate.

There are also examples in which the evolutionary rise of a new sex determination system is inconsistent with the pre-conditions of any of the directional selection hypotheses. Multiple male-determiners, for instance, that co-exist in the dipteran insects *M. domestica* and *M. scalaris* 10, 35, 85, cannot be used to adjust sex ratios, nor do different male-determiners promote sexual conflict. It is not plausible that evolutionary degeneration of a preceding sex determiner (*i.e*. through the accumulation of deleterious mutations) would directionally select for multiple male sex determiners. Fitness differences of *M. domestica* populations that either have an autosomal *M* or a *Y-M* sex determination mechanism 86 can also be explained by secondary effects and genetic differences associated with X/Y chromosomal backgrounds.

Another explanation for the evolutionary rise of alternative male-determiners is genetic drift. A higher evolutionary origin rate by mutation will increase the probability of fixation (see 87 for a theoretical analysis of how genetic drift can drive genetic pathway evolution). Multiple male-determiners (>4) in *M. domestica* and *M. scalaris* populations could reflect the increased mutation rate by which new repressing activities of *tra* genes can evolve from the entire gene repertoire of the genome. A repressing activity can be established at different levels of *tra* gene regulation, suggesting a high rate of origin. In contrast, female-determiners evolve more rarely (indeed, only one female-determiner has been identified in *M. domestica*, but none in *M. scalaris*) because it is less likely that a new function arises by mutation that properly controls *tra* pre-mRNA splicing and protein production.

The null-hypothesis of genetic drift has been rejected in the case of the *csd* gene. An excess of non-synonymous over synonymous, neutral nucleotide changes has been identified during the evolutionary origin of *csd* (Fig. 3B) 12. More amino acid-encoding nucleotides have been replaced directly after *csd* origin than neutral changes that solely became fixed by random genetic drift. Intriguingly, some of these new amino acids form a putative coiled-coil domain that appears to alter binding properties between Csd allelic proteins (Otte and Beye, unpublished results). The cause of directional selection during the evolutionary rise of *csd* is not understood and cannot be explained by an adjustment of sex ratios or an intra-locus sexual conflict. Complementary sex determination systems allow control over sex ratios (males derive from unfertilised eggs, females from fertilised eggs) and are not targets of intra-locus sexual conflict and sex ratio drive (as they are inherited in both sexes). Complementary sex determination systems can reside in genomic regions of substantially-reduced meiotic recombination 70, 88, implying that evolutionary degeneration may operate. Malfunction of the preceding complementary sex determiner could have selected for a new sex determiner; *csd*.

## Fruitful routes of future research

There is a need to characterise more sex determination genes. Discovering other regulatory mechanisms and their routes of evolutionary origin will broaden our understanding of how and why these key controls of developmental processes have evolved. Characterisation of sex determiners will be greatly facilitated by next-generation sequencing technologies, especially for species in which sex-determining factors can be linked to short genomic regions of otherwise freely-recombining chromosomes (*e.g*. neo Y chromosomes) 9, 10, 31, 35, 85, 89. Sequencing DNA from pools of male and female progenies of a single cross will identify sex-specific nucleotide differences. These sex-specific differences will identify candidate genes. The function of these genes can be further tested using RNAi-induced knock-down studies.

Nucleotide sequences of novel sex determiners will allow us to trace the evolutionary history 12, 15, 69 and the evolutionary forces that shaped their origin 12. By applying population genetic tests on nucleotide sequences (*i.e*. dN/dS ratio, MacDonald Kreitman test, Tajima's *D*, among others 90), we can directly identify evidence for the cause of evolutionary change 12 in the responsible genes. This powerful approach has been widely neglected in evolutionary developmental biology.

An open question is when did the key sex-determining functions of *tra* (Figs. 1, 3) evolve? *tra* genes apparently have no sex-determining function outside of insects (see the crustacean case *Daphnia magna* 91), but it is not known whether hemimetabolous insects use these *tra* key functions. The fast-evolving *tra* genes may be identified by a conserved 30 amino acid motif that has been identified in non-*Drosophila* species 12, 13, 15, 92, 93. *tra* has not been identified in the sequenced *B. mori* genome 94, 95, which may reflect a lack of sufficient conservation, evolutionary loss, or a lack of corresponding sequence information.

What is widely unknown is how sexual dimorphic traits can evolve so rapidly by changes in the underlying developmental program. Reproductive structures, behaviours and secondary sexual characteristics are some of the most variable and changeable features among insects; some of which evolve through sexual selection (Darwin 1871). Morphological changes may evolve by the regulatory control of *dsx* target genes [by either modification, loss or novel origin of *cis* regulatory elements (CREs) 52] or by recruiting other genes (*i.e*. transcription factors) to the sex-determining process. Investigation into what aspects of morphology *dsx* controls in different species could give insights into whether *dsx* is a conserved key determinant of sexual dimorphic differentiation. Transgenic studies have shown that at least some aspects of *dsx* sexual differentiation are conserved in the dipteran insect *M. domestica* 16 and the lepidopteran *B. mori* 55, 96. Expression of male and female Dsx proteins, with the help of transgenic tools in a *dsx* null mutant background, would facilitate study into which aspects of sexual differentiation are controlled by *dsx* (RNAi-induced knockdown of *dsx* by injection procedures have, thus far, failed in several insects) and whether other key components in non-*Drosophila* species have yet to be identified. The evolution of abdominal pigmentation and pheromone production in some *Drosophila* species have been shown to be caused by small-scale evolutionary changes of CREs of Dsx protein target genes 52, 63, 97. A way to identify putative shared and evolved *dsx* target genes is to characterise binding sites of Dsx proteins in informative insect species by chromatin immuno-precipitation (ChiP) and next-generation sequencing.

## Conclusion

Recent studies in insects have shed some light on how and why sex determination systems evolve 1, 12, 15, 69. Gain-of-function alleles of *tra*, the double dose of X-linked transcription factors that activate *Sxl*, or Csd proteins derived from a heterozygous *csd* gene are the molecular signals of diverse sex determination mechanisms, such as a dominant female-determining system, an X:A ratio and a complementary sex determination system. These organism-specific signals all share transduction of their activities to common *tra* genes; the upstream component of an ancestral pathway tra->dsx in holometabolous insects. Diverse signals utilise two regulatory principles to determine the two sexes (Fig. 2): they either produce feminising activities that switch *tra* genes ON, or they produce masculinising activities that switch *tra* genes OFF. In the absence of these activities, the pre-zygotic activity of *tra* (‘default state’ either ON or OFF) executes male or female development. *tra* genes in most studied insects maintain the sexually-determined state epigenetically through a positive regulatory feedback loop of pre-mRNA splicing 13.

Diverse mechanisms can evolve through small-scale nucleotide changes in regulatory and coding regions from existing or from duplicated genes. From the ease and rate that novel sex determiners arise by such changes 12, even within a species 10, 15, 35, 85, we suggest that non-adaptive forces (mutation and genetic drift) are also possible sources of novel sex determination systems. We suggest that genetic drift should serve as a null hypothesis in future work.

An excess of non-synonymous changes over synonymous, neutral changes 12 in the *csd* gene of honeybees shows that directional selection can enhance the evolutionary rise of novel sex determiner genes 12. Evolutionary degeneration, due to lack of recombination of the preceding complementary sex determiner gene, may have caused such directional selection 6, 12, 82, 83.

By applying molecular evolutionary and genetic approaches, we are just beginning to understand both the evolutionary routes and the molecular mechanisms that have generated the enormous diversity of sex determination mechanisms. These insights will greatly broaden our knowledge of how novel control mechanisms and regulatory principles evolve in developmental processes.
